# Chronic Palmitate Exposure Inhibits Insulin Secretion by Dissociation of Ca^2+^ Channels from Secretory Granules

**DOI:** 10.1016/j.cmet.2011.03.002

**Published:** 2011-04-06

**Authors:** Michael B. Hoppa, Stephan Collins, Reshma Ramracheya, Leanne Hodson, Stefan Amisten, Quan Zhang, Paul Johnson, Frances M. Ashcroft, Patrik Rorsman

**Affiliations:** 1 The Oxford Centre for Diabetes, Endocrinology, and Metabolism, Churchill Hospital, Oxford OX3 7LJ, UK; 2 Nuffield Department of Surgery, John Radcliffe Hospital, Oxford OX3 9DU, UK; 3 Department of Physiology, Anatomy, and Genetics, Oxford University, Oxford OX1 3PT, UK

The study was supported by funding from the NIHR Oxford Biomedical Research Centre program.

